# Diagnosing cystic duct patency during myocardial perfusion imaging (MPI), using Tc99m Sestamibi (MIBI), as an adjunct benefit in the acute setting

**DOI:** 10.1259/bjro.20200008

**Published:** 2020-12-21

**Authors:** Hassan Semaan, Haitham Elsamaloty, Mohamad Bazerbashi, Joud Obri, Mazzin Elsamaloty, Alberto J Arroyo, Tawfik Obri

**Affiliations:** 1 Department of Radiology, University of Toledo Medical Center, Toledo, OH, USA; 2 Department of Radiology, Nuclear Medicine, St. Vincent Mercy Medical Center, Toledo, OH, USA

## Abstract

**Objective:**

Tc99m methoxy isobutyl isonitrile (MIBI) has been used for myocardial perfusion imaging (MPI) for the detection of ischemia. This study aimed to investigate the feasibility of effectively evaluating cystic duct patency, during routine visual analysis of the raw MPI and/or with the three-dimensional reconstructed data.

**Methods:**

A retrospective investigation of 91 patients undergoing cardiac MIBI scan for acute chest pain and hepatobiliary scintigraphy (HBS) was performed, within no more than 3 months for suspected gallbladder obstructive disease. Gallbladder visualization during either the stress or rest portion of the MIBI was indicative of cystic duct patency. These results were compared to those by the HBS studies.

**Results:**

Ten patients had the MIBI and HBS 4 days apart, both analyses concurred 100% with the diagnosis of cystic duct patency. 16 patients had both examinations between 4 days and 3 weeks and had an agreement of 87.5% with cystic duct patency. 65 patients had both tests 3 weeks to 3 months apart and had an agreement of 84.6% with cystic duct patency.

**Conclusion:**

The initial results of this study indicate that MPI with Tc99m MIBI is useful in detecting a patent cystic duct, above all in the setting of acute gallbladder pathology.

**Advances in knowledge:**

In this article, we introduce a novel method to diagnose cystic duct patency in the acute setting thus effectively ruling out acute cholecystitis, during MPI. Our method can potentially improve patient outcomes by reducing the volume of imaging needed to exclude a diagnosis of acute gallbladder pathology. This in turn, keeps in line with decreasing the cost for the patient, leading to a more sound value-based care.

## Introduction

Tc99m methoxy isobutyl isonitrile (MIBI) has been used for myocardial perfusion imaging (MPI) for the detection of ischemia in patients with known or suspected coronary artery disease (CAD). Similarly, Tc99m labeled hepatic imino diacetic acid (HIDA) derivatives have been used to evaluate gallbladder (GB) disease, which is one of the most common digestive system illnesses around the world. It would not be unusual when an emergency hepatobiliary scintigraphy (HBS) is ordered a few hours after the MPI is finished or a day later. Since the early1990’s, patients have been undergoing cardiac MIBI scans to assess myocardial perfusion in coronary artery disease. This test uses the single photon emission computed tomography (SPECT) to give the interpreting physician a better look at the perfusion of the myocardium. MIBI is taken up physiologically by the heart, salivary glands, thyroid, liver, and spleen. The tracer is excreted through the renal and the hepatobiliary systems. The hypothesis of our study is to determine if we could effectively diagnose cystic duct patency in the acute setting, while using MIBI to evaluate myocardial perfusion. There have been other various pathological incidental findings/reports in the literature when patients undergo cardiac studies with MIBI.^1–4^ Any additional findings from the review of the raw data in cine mode should be routinely included in cardiac SPECT MIBI reports, to prompt the referring physician to pursue further investigations, since they may have clinical impact. By contrast, the present study with MIBI alone, evaluates the presence of a patent cystic duct, essentially ruling out acute cholecystitis, which can be extremely useful in the acute setting. As the MIBI and HBS are performed within close proximity of each other on the same patient, a direct comparison of the results can be assessed. Since the introduction of Tc99m-IDA cholescintigraphy in the late 1970’s, which has similar physiology as MIBI with regards to the liver and GB, we have been able to further gather functional information concerning the patency/obstruction of the cystic duct in patients with right upper quadrant pain.^5^ However, to our knowledge no other research paper in the literature has compared MIBI GB visualization and corresponding HBS imaging on the same patient. If the interpreting and referring physicians were made aware of the benefits of being able to diagnose cystic duct patency in the acute setting, while using MIBI to evaluate myocardial perfusion, it would lead to earlier diagnosis and more efficient patient care; thereby, decreasing the amount of imaging that patients need to undergo while being assessed for acute GB pathology.

## Methods and materials

**Figure 1.**
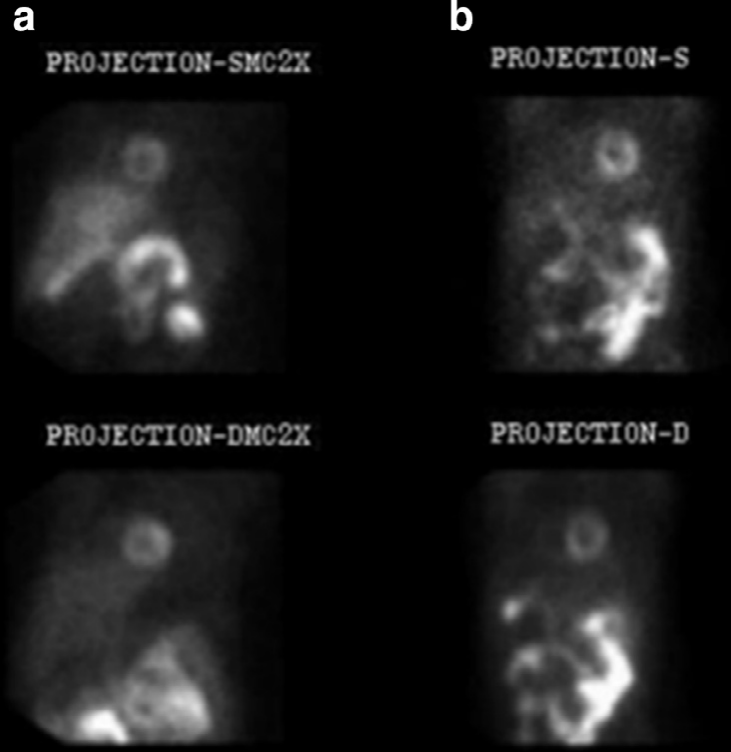
GB visualized on stress only (a) and rest only (b) MIBI projection data. GB, gall bladder; MIBI, methoxy isobutyl isonitrile.

The study was approved by our institutional review board committee and patients’ informed consent was waived. Over a 2-year period of retrospective data analysis; from Jan 1, 2014 to Dec 31 2015, 2950 total MIBI’s were acquired (80.3% pharmacological, and 19.7% treadmill studies), and 91 of those patients (3.1%) also had HBS evaluation. 58 patients were females (64%) and 33 patients were males (36%). Average age was 57 years (range 25–89). Average body mass index (BMI) was 37 (range 16–46). The average number of days the HBS study was performed after the MIBI was 36 days (range 3–89). The 91 patient population were categorized into three groups based on the time period between the MIBI and HBS imaging; Group 1 included patients who had both examinations acquired within a 4-day period, Group 2 included patients who had both scans performed within 4 days to 3 weeks, and Group 3 included patients who had both protocols imaged >3 weeks to 3 months apart. GB visualization during either the Stress or Rest portion of the MIBI was indicative of cystic duct patency (Figure 1). These results were then compared to those obtained by the HBS. Current SNMMI procedural standards for myocardial perfusion imaging (MPI)^6^ and HBS^7^ were strictly followed. Data were acquired using a LFOV dual-head (ADACForte Gamma Camera, Philips) ADAC was acquired by Philips Medical Systems, Cleveland Ohio 44143 camera fitted with high resolution collimators. All data collected were on hospitalized patients. Typical MPI protocol consisted of a minimum 4 h fasting state, with cardiac medications that interfered with CAD detection being withheld. Caffeine containing food/beverages and methylxanthine containing medication were also withheld for a minimum of 12 h. For a 1-day protocol, 11–13 mCi was used for the rest MPI. The dose was adjusted based on decay for the stress portion. Imaging was started 45 min post-injection, during which time patients usually had coffee and cookies. For a 2-day protocol, 25–30 mCi was used. Typical HBS protocol consisted of a minimum fasting of 4–7 h and no opiates for at least 24 h. 3–5 mCi of Mebrofenin was used and imaged for 1 h. Morphine Sulfate may be given to differentiate chronic *vs* acute cholecystitis.

## Results

**Figure 2.**
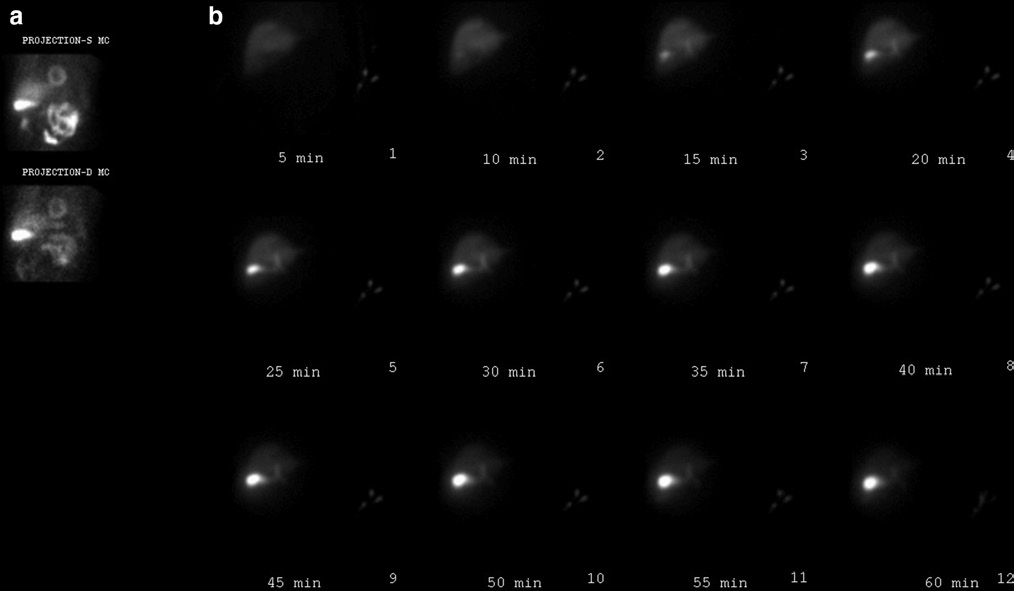
GB visualized on the stress/rest MIBI (a) and HBS data (b). GB, gall bladder; HBS, hepatobiliary scintigraphy; MIBI, methoxy isobutyl isonitrile.

**Figure 3.**
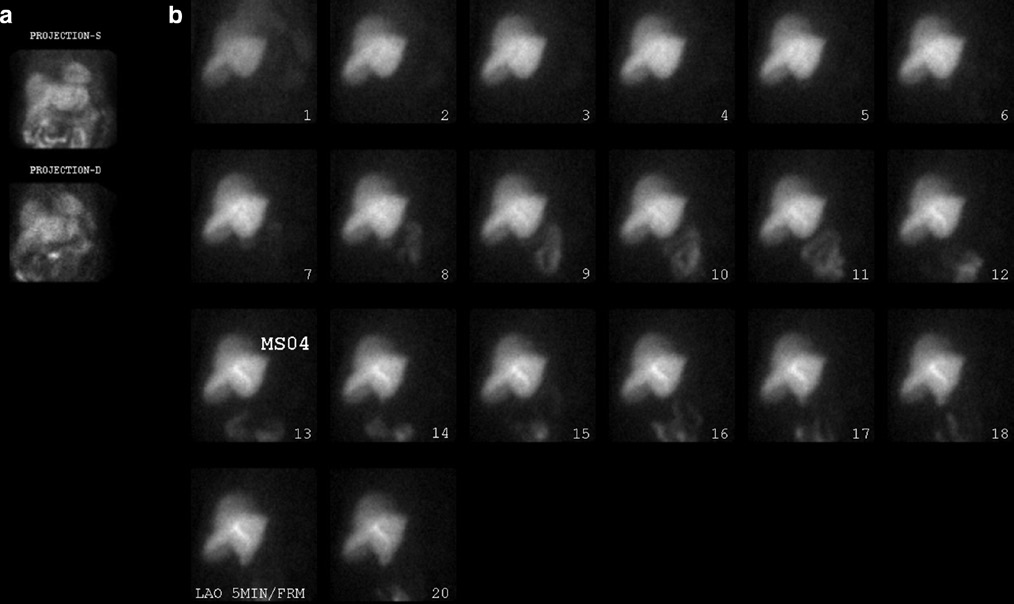
GB not visualized on either the stress/rest MIBI (a) or HBS post MS04 data (b). GB, gall bladder; HBS, hepatobiliary scintigraphy; MIBI, methoxy isobutyl isonitrile.

**Figure 4.**
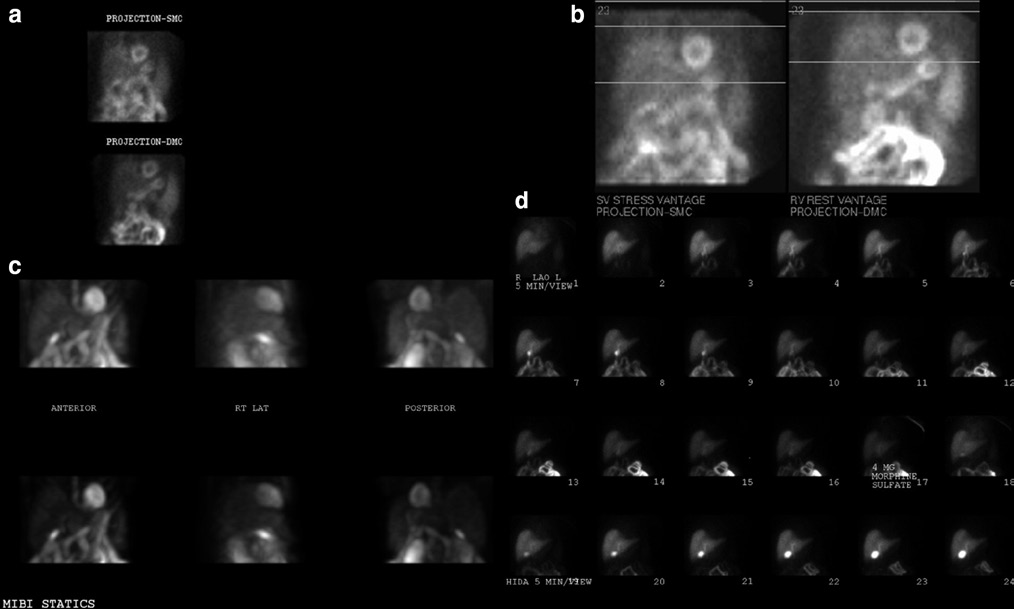
(a) Faintly visualized GB on the stress/rest MIBI data. (b) Enlarged S/R data with different intensity, not convincing enough. (c) Ant., Rt. Lat. and Post. Frames of the 3D reconstructed Stress data, clearly shows the GB. The 3D data in Cine Mode, gives an unequivocal GB visualization in its proper location. (d) HBS shows what appears to be GB at frames 5–9; the Cine Mode reveals passage of the radiotracer through the “C” loop of the duodenum. Post MS04 at frame #17 and a booster dose, the GB appears, in the same location as the 3D data. 3D, three-dimensional; GB, gall bladder; HBS, hepatobiliary scintigraphy; MIBI, methoxy isobutyl isonitrile.

Group 1 Included 10 patients (5 males and 5 females, age 60.2 ± 18.6 years and a body mass index (BMI) 31.9 ± 11.4), who had the MIBI and HBS 4 days apart. In 5 patients out of 10 (50%), both MIBI and HBS resulted in GB visualization and therefore concurred with the diagnosis of no acute cholecystitis (Figure 2). In 3 patients out of 10 (30%), both MIBI and HBS showed no visualization of GB and concurred with the diagnosis of acute cholecystitis (Figure 3). In 2 patients out of 10 (20%), both MIBI and HBS disagreed with the diagnosis; while MIBI did not indicate acute cholecystitis, HBS suggested chronic disease (Figure 4). The agreement rate in this group was 80%, while the disagreement rate was 20%. However, this group had a 100% agreement with cystic duct patency. In these 10 patients, the MIBI study was always performed first, followed by the HBS which was usually ordered a few hours later or a whole day later.

Group 2 included 16 patients (5 males and 11 females, age 59.6 ± 14.3 years, and BMI 31.7 ± 11.2), with both studies from 4 days to 3 weeks apart. Both MIBI and HBS scans were negative and concurred with a normal GB in 14 patients (87.5%). In two patients (12.5%), MIBI and HBS disagreed with the diagnosis. While MIBI proposed acute cholecystitis, HBS suggested chronic disease. The agreement rate in this group was 87.5%, while the disagreement rate was 12.5%. Nevertheless, this group had an 87.5% agreement with cystic duct patency.

Group 3 Included 65 patients (42 males and 23 females, age 56.3 ± 12.9 years, and BMI 34 ± 12) with both MIBI and HBS 3 weeks to 3 months apart. In 52 patients (80%), both series concurred with no acute cholecystitis. In 3 patients (4.6%), both protocols concurred with acute cholecystitis. MIBI and HBS disagreed with the diagnosis in 10 patients (15.4%); in 3 patients (4.6%), while MIBI proposed no acute disease, HBS diagnosed a chronic condition, in 4 patients (6.2%), MIBI suggested acute cholecystitis, while HBS indicated chronic disease, and in 3 patients (4.6%), MIBI showing a normal result compared to an acute disease on the HBS. The agreement rate in this group was 84.6%, while the disagreement rate was 15.4%. Then, again both studies had 84.6% agreement with the cystic duct patency. Of the combined groups, 7 on Group 1, 14 on Group 2 and 55 on Group 3, the MIBI and HBS procedures concurred with the presence of a patent cystic duct. This gave an overall agreement of 100% for a total of 76 patients.

### Statistics

ERCP is currently the gold-standard for the diagnosis of biliary obstruction, albeit one of several invasive direct cholangiography techniques, and since neither MIBI nor HBS can be considered as the gold-standard, then statistics such as sensitivity, specificity, etc. are not appropriate. However, we can measure the strength of the agreement between the results from the MIBI and HBS, agreement beyond what is expected by chance alone, with κ statistics. In this investigation, the κ coefficient is 0.42 with 95% confidence interval 0.16–0.69.^8,9^

## Discussion

**Figure 5.**
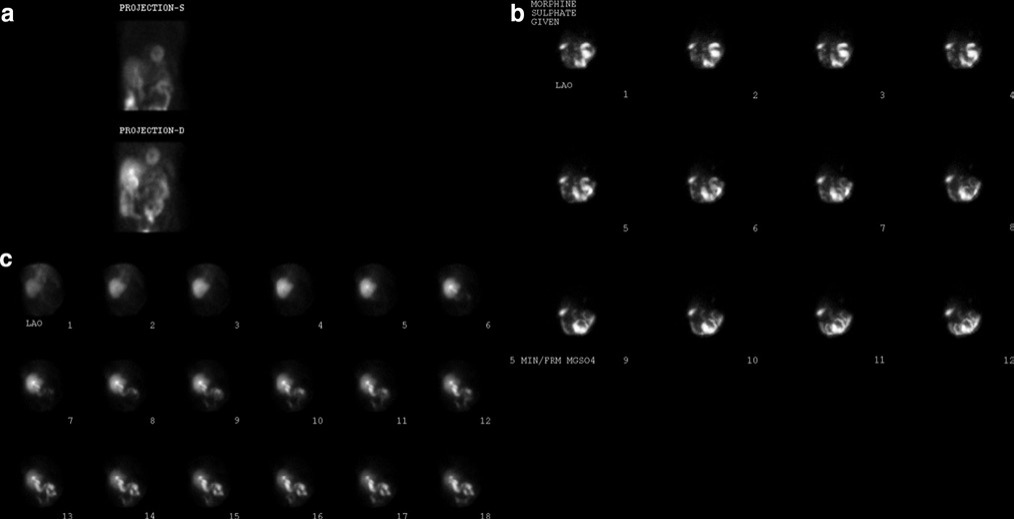
GB not visualized on the stress/rest MIBI (a) or HBS data (b), except post MS04 (c). *Incidentally, the MIBI, pre-MS04 and post-MS04 HBS studies were acquired in three different γ cameras*. GB, gall bladder; HBS, hepatobiliary scintigraphy; MIBI, methoxy isobutyl isonitrile.

**Figure 6.**
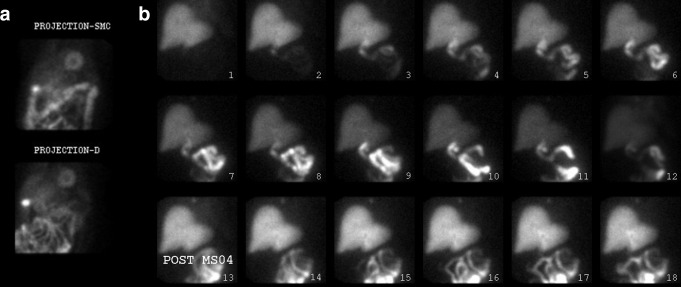
GB visualized on the stress/rest MIBI (a), not on the HBS data even post MS04 and a booster dose (b). GB, gall bladder; HBS, hepatobiliary scintigraphy; MIBI, methoxy isobutyl isonitrile.

Tc99m MIBI is a cationic compound that accumulates in the myocardium in proportion to blood flow. Uptake is high in muscle, liver and kidneys, with a lower uptake in the thyroid and salivary glands.^10^ Its elimination is mostly through the kidneys (27%) and the hepatobiliary system (33%) into the gastrointestinal tract.^11^ Tc99m MIBI appears to passively diffuse into the hepatocytes, and its biliary excretion is mediated by a plasma glycoprotein,^12^ with maximum GB accumulation occurring approximately 30–60 min post-injection, provided a patent cystic duct is present. Based on this study’s results, there was 100% agreement in 76 patients (83.5%) for cystic duct patency evaluation. Of the remaining 15 patients (16.5%), 9 were diagnosed with acute cholecystitis by the HBS protocol, and the other 6 were diagnosed as having chronic cholecystitis by the use of either Morphine Sulfate (MSO4) or Sincalide, a synthetic C-terminal octapeptide of cholecystokinin (CCK-8). Once again, in general, none of these pharmaceutical agents could be used during a MIBI protocol. The results of those 15 patients require the following explanation. A patient may already be NPO from midnight on, expecting evaluation and/or a cardiac consult, for an assessment with MIBI. If the order for the MPI comes too late in the day, there may not be enough time to do the “rest” portion first. If the “stress” portion is performed first, the patient may already be on a “prolonged fasting” state (which may require pre-treatment with CCK-8 in the HBS protocol), and this may prevent the GB from visualizing with MIBI. The “rest” portion is then performed 3 to 4 h later (if the “stress” portion is performed first). Whether the patient eats or not, the MIBI may still be unable to visualize the GB, due to the same physiological conditions that occur on an HBS, for which the optimal fasting time is 4–7 h. In any case, in the HBS protocol if there is any doubt, pretreatment with CCK-8 is performed to avoid false-positive results. And, this may be the reason why in 12 of the 91 MIBI’s the GB failed to visualize, implying that the cystic duct was not patent and hence suggesting a diagnosis of acute cholecystitis, obviously false-positive results, while the HBS studies on those 12 patients were diagnosed with either chronic cholecystitis or normal (Figure 5). 3 patients out of the 91 were challenging in which the MIBI demonstrated a clearly visualized GB indicating a patent cystic duct, as a result excluding acute cholecystitis. However, the HBS failed to visualize the GB and were diagnosed with acute cholecystitis. All three patients were from Group 3. Further investigation of the data revealed that the MIBI was done first in all 3 cases and they were between 8 and 11 weeks apart. Incidentally, 2 of the 3 patients were allergic to MSO4, and were imaged at the 5 h mark. We postulate that it is possible these three patients had a chronic condition of the GB, and by the time they came to have a HBS scan, and their problem became worse, the HBS protocol diagnosed them as having acute cholecystitis (Figure 6), indicating that the long interval appearance of biliary disease would be the more likely cause. Table 1 demonstrates that the earliest period an HBS can be performed post R/MIBI study is 4 days later. In the occasional presentation of chest pain followed by right quadrant pain in the weekend as demonstrated; a GI/Surgical consult requesting a STAT HBS, if willing to accept a diagnosis of “patent cystic duct” from such a MIBI data may help eliminating the second examination (HBS). It also demonstrates the maximum length between both protocols if the orders come down over the weekend. One may argue that there is no need to wait 48 h between MPI and HBS, however in most cases the GB will still be well visualized at 24 h. from the MIBI study.

**Table 1.** Time period between ordering MBI and HBS studies

Time/Day	1 Saturday	2 Sunday	3 Monday	4 Tuesday	5 Wednesday
AM			S/MIBI	Clearance & decay	HBS
PM	R/MIBI	STAT HBS ordered			

HBS, hepatobiliary scintigraphy; MIBI, methoxy isobutyl isonitrile..

### Limitations

The limitations of the present study include its retrospective nature as well as the relatively small sample size. Furthermore, proper evaluation of possible chronic cholecystitis, by the use of MSO4, or CCK-8, was not feasible. The only exception would be the use of CCK-8 in those very extraordinary cases where the GB lies in the field of view of the myocardium as the MIBI data are being acquired. Moreover, acute cholecystitis evaluation was not reliable as the MIBI protocol does not adhere to the optimal fasting time of 4–7 h. as in the HBS protocol. Patients with a 1 day or 2 day MIBI protocol are already in a prolonged fasting state or may be allowed to eat a light meal before the “rest” portion of the MPI. Finally, we were not able to estimate how long a negative MIBI (visualization of the GB indicating a patent cystic duct), might be reliable to document cystic duct patency and as a consequence essentially exclude acute cholecystitis, with the possible exception of Group 1, which is 4 days. Such estimation is to a large extent limited by the greater temporal gap between MPI and HBS scans, such as in Groups 2 and 3.

## Conclusions

The initial results of the present study, although in a small number of patients, especially for the acute setting of Group 1 (10 patients), indicate that MPI with Tc99m MIBI is useful in effectively detecting a patent cystic duct and therefore essentially excluding acute cholecystitis. The results showed 100% agreement with those obtained with the HBS protocol in a total of 76 patients. And more interestingly, as we increased the time interval between both protocols, and the patient sample size along with it, the agreement rates with cystic duct patency decreases to 87.5% for the group of 3 weeks interval and to 84.6% for the group of up to 3 months interval, although the agreement and disagreement rates remained relatively constant. In conclusion, this investigation may not only add incremental data to the body of literature on the scintigraphic imaging of GB disease, it also should prove to be a useful addition for GB evaluation, especially in the acute setting and ought to help in eliminating unnecessary additional testing and corresponding radiation exposure.

### Advances in knowledge

In this article, we introduce a novel method to diagnose cystic duct patency thus effectively ruling out acute cholecystitis in the acute setting, while using MIBI to evaluate myocardial perfusion. Our method can potentially improve patient outcomes by reducing the volume of imaging needed to reach an earlier diagnosis of cystic duct patency. This in turn, keeps in line with decreasing the cost for the patient, leading to a more sound value-based care.
